# Proteome analysis of NRF2 inhibition in melanoma reveals CD44 up‐regulation and increased apoptosis resistance upon vemurafenib treatment

**DOI:** 10.1002/cam4.4506

**Published:** 2021-12-23

**Authors:** Hans Peter Weitzenböck, Anna Gschwendtner, Christoph Wiesner, Maren Depke, Frank Schmidt, Franz Trautinger, Markus Hengstschläger, Harald Hundsberger, Mario Mikula

**Affiliations:** ^1^ Medical and Pharmaceutical Biotechnology IMC University of Applied Sciences Krems Austria; ^2^ Center for Pathobiochemistry and Genetics Medical University of Vienna Vienna Austria; ^3^ Interfaculty Institute for Genetics and Functional Genomics University Medicine Greifswald Greifswald Germany; ^4^ Proteomics Core Weill Cornell Medicine‐Qatar Qatar Foundation‐Education City Doha Qatar; ^5^ Department of Dermatology and Venereology University Hospital of St. Pölten Karl Landsteiner University of Health Sciences St. Pölten Austria; ^6^ Karl Landsteiner Institute of Dermatological Research St. Pölten Austria; ^7^ Department of Dermatology University Hospital of the Paracelsus Medical University Salzburg Austria

**Keywords:** anti‐oxidant response, combination therapy, malignant melanoma, redox homeostasis

## Abstract

Malignant melanoma is the deadliest form of skin cancer and NRF2 has been proposed as a main regulator of tumor cell malignancy. Still the mechanisms how NRF2 is contributing to melanoma progression are incompletely understood. Here we analyzed the effects of either NRF2 induction or depletion, and we also quantified changes on the whole cell proteome level. Our results showed that inhibition of NRF2 leads to a loss of reactive oxygen species protection, but at the same time to an induction of an epithelial mesenchymal transition (EMT) phenotype and an up‐regulation of the stem cell marker CD44. Additionally, cells devoid of NRF2 showed increased cell viability after treatment with a MYC and a BRAF inhibitor. Importantly, survival upon vemurafenib treatment was dependent on CD44 expression. Finally, analysis of archival melanoma patient samples confirmed a vice versa relationship of NRF2 and CD44 expression. In summary, we recorded changes in the proteome after NRF2 modulation in melanoma cells. Surprisingly, we identified that NRF2 inhibition lead to induction of an EMT phenotype and an increase in survival of cells after apoptosis induction. Therefore, we propose that it is important for future therapies targeting NRF2 to consider blocking EMT promoting pathways in order to achieve efficient tumor therapy.

## Graphical abstract

Down‐regulation of NRF2 in melanoma led to an epithelial mesenchymal transition phenotype and an up‐regulation of the stem cell marker CD44. Additionally, cells devoid of NRF2 showed increased cell viability after treatment with a MYC and a BRAF inhibitor, but lost their protection against reactive oxygen species‐derived stress.

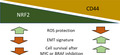

## 1 INTRODUCTION

The main regulator of cellular anti‐oxidant response is NRF2. It is a transcription factor driving expression of genes involved in redox homeostasis, drug metabolism, energy metabolism, survival, proliferation, DNA repair, and others.^1^, ^2^ The availability of NRF2 is under the control of E3 ubiquitin ligases, with the Keap1‐Cul3‐RBX1 complex as the dominant regulator.^3^ Mechanistically, oxidative stress reduces the binding ability of KEAP1 to NRF2, therefore NRF2 is not degraded, but cellular amounts increase and drive specific target gene expression.

The role of NRF2 in tumor progression is complex. While NRF2 activation can prevent radiation‐ and chemical‐induced tumor onset, NRF2 has also been described to promote cancer progression.^4^ In hepatocellular carcinoma and in breast cancer this phenomenon has been attributed to the pro‐proliferative effect of NRF2 activation and the expression of its target genes in cancer cells.^5^, ^6^ In pancreatic cancer increased protein translation has been monitored by NRF2 dependent stabilization of mRNAs.^7^ Moreover, oncogenic mutations in KRAS and BRAF increase the amount of NRF2.^8^, ^9^

The human skin is exposed to a plethora of environmental factors, which can cause cellular stress and are involved in triggering cancer formation. In most human melanomas the NRF2 system is not constitutively activated, as mutations in NRF2 and KEAP1 are only sporadic.^10^, ^11^ Importantly, it has been shown that enhanced expression of NRF2 in melanoma cells worsened patient survival.^12^ Hence, it seems plausible that NRF2 could represent a valuable target in anti‐tumor therapy. In order to elaborate on this question, we screened for molecular signatures on the proteome level in melanoma cell lines. As expected we identified reactive oxygen species (ROS) pathway up‐regulation in NRF2 induced cells. Surprisingly, we identified enrichment of an EMT‐signature in NRF2 depleted cells, which was hallmarked by up‐regulation of CD44. CD44 is a stem cell biomarker enhancing cancer cell invasiveness, chemoresistance and EMT‐properties.^13^ Importantly, cell killing by MYC inhibition or vemurafenib treatment was less effective when NRF2 was down‐regulated. Furthermore, analysis of human melanoma samples confirmed a vice versa relationship of NRF2 and CD44 expression.

To the best of our knowledge, this is the first report analyzing NRF2 targets on the proteome level in melanoma. Our findings propose that targeting of NRF2 in melanoma could lead to CD44 up‐regulation, which enhances survival of melanoma cells.

## 2 MATERIAL AND METHODS

### 2.1 Cell lines and culture

Non‐metastatic MCM1G and metastatic MCM1DLN lines where isolated from a human melanoma patient and characterized.^14^ Non‐metastatic WM793b and metastatic WM1205Lu (termed 1205LU) lines were obtained from ATCC.

Cells were cultivated at 37°C with 5% CO_2_ in melanoma isolation media (MIM). MIM consisted of three parts MCDB153 medium (M7403, Sigma‐Aldrich) with one part Leibovitz's L‐15 medium (L5520, Sigma‐Aldrich) and was supplemented with 2% FBS, 7.5% NaHCO_3_, 1.68 mM CaCl_2_, 5 μg/ml Insulin and 5 ng/ml epidermal growth factor. Cells were split when 90% confluency was reached, using a 0.25% trypsin/EDTA solution for detachment.

### 2.2 Transient knockdown by siRNA

Reagents for siRNA treatment, containing four individual siRNAs per target, were obtained from Dharmacon (SMARTpool, L‐003755‐00, Dharmacon).^15^ A set of nonspecific non‐targeting siRNA sequences (NT) were included as negative control, undergoing the same transfection procedure. Recommended procedure was followed. Briefly, the SMARTpool siRNA mixture consisting of four individual siRNAs is applied to cells at a final concentration of 25 nM with a ratio of 2.5–1 to DharmaFECT 1 Transfection Reagent (T‐2001‐03, Dharmacon). Cells were transfected in MIM without antibiotics. Cells were used for RNA isolation 24 h after transfection and for protein extraction or further culture experiments 48 h after transfection.

### 2.3 Immunofluorescence imaging and quantification

After overnight cultivation, cells were fixed with 4% formaldehyde for 15 min and quenched by 100 mM glycine for another 15 min. Cell walls were permeabilized by 0.2% Triton for 15 min and blocked with 1% BSA for 1 h at room temperature. NRF2 antibody (1:500, ab62352, Abcam) and 1% BSA were applied at 4°C overnight. Secondary antibody Alexa Fluor 488 anti‐rabbit goat IgG (1:800, A‐11001, Thermo Fisher Scientific) was applied at room temperature for 1 h in the dark. Vectashield mounting medium containing DAPI (H1500, Vector Laboratories) was applied. Imaging was performed on a Leica TCS SP8 confocal microscope (Leica Camera). Overlays and color intensity quantification were conducted in Adobe Photoshop 2021.

### 2.4 Western blot

Protein samples were isolated from cell culture using RIPA buffer (9806S, New England Biolabs) and quantified by Bradford assay (7780S, New England Biolabs). 20 µg of protein were boiled in Laemmli buffer (1610747, Bio‐Rad Laboratories) at 95°C for 10 min and loaded onto 4%–20% Mini‐PROTEAN^®^ TGX Stain‐Free™ Protein Gels (4568096, Bio‐Rad Laboratories). Proteins were blotted in a Trans‐Blot Turbo Transfer System (Bio‐Rad Laboratories) using Trans‐Blot^®^ Turbo™ Mini Nitrocellulose Transfer Packs (1704158, Bio‐Rad Laboratories). Membranes were blocked using a 5% dried milk solution (9999S, New England Biolabs). Antibody solutions were kept in 1% milk solution with 0.01% Sodium Azide. Antibodies were used to target NRF2 (1:500, ab62352, Abcam), KEAP1 (1:500, 10503‐2‐AP, Proteintech Group), NADP‐dependent malic enzyme (ME1, 1:200, sc‐100569, Santa Cruz Biotechnology), Glutathione reductase (GSR, 1:100, sc‐133136, Santa Cruz Biotechnolo‐gy), CD44 (1:2000, 15675‐1‐AP, Proteintech Group) and Beta Actin (1:200, Sc‐47778, Santa Cruz Biotechnology). Secondary antibodies were IRDye^®^ 800CW Goat against rabbit (926‐32211, LI‐COR Biosciences) and mouse (926‐32210, LI‐COR Bio‐sciences). Primary antibodies were applied at 4°C overnight, secondary antibodies at room temperature for 1 h. Images were recorded on a ChemiDoc MP system (Bio‐Rad Laboratories) and processed and quantified in ImageLab software (Bio‐Rad Laboratories).

### 2.5 Quantitative PCR

RNA samples were obtained from cell culture using the RNeasy Kit (74106, Qiagen), following the manufacturers standard protocol. Synthesis of cDNA was performed with qScript cDNA SuperMix (95048, Quantabio) following DNase I treatment (M0303S, New England Biolabs). TAQMAN primer pairs and probes were designed for genes of interest and ordered from Sigma Aldrich (VC00021, VC00023, Sigma‐Aldrich). Design parameter for primers were size between 18–25 bp, melting temperature between 57–62°C and a product size range of 80–150 bp. Design parameter for probes were size between 18–30 bp and a melting temperature between 67–72°C. The probe was equipped with 6‐FAM (5’) and BHQ1 (3’). For running the qPCR TaqMan^®^ Gene Expression Master Mix (4369514, Thermo Fisher Scientific) was used with 6 µM of primers and 2 µM of probe. Runs were carried out on a QuantStudio 7 Flex (Thermo Fisher Scientific) device, with 45 cycles of 95°C for 20 s and 60°C for 40 s. Relative differences were calculated by applying the ∆∆cq Method, using EEF1A1 as housekeeping gene for normalization.

### 2.6 Reactive oxygen species quantification

ROS in the cells was measured by 2′, 7′‐Dichlorofluorescin diacetate (DCFDA) assay. After overnight cultivation they were loaded by applying 20µM DCFDA to antibiotic free culture medium at 37°C for 30 min. Cells were washed and treated with 10 mM H_2_O_2_. DCFDA was measured in a SpectraMax i3x Multi‐mode microplate reader (Molecular Devices) at excitation/emission wavelengths of 485/535 nm. Measurements were taken over a time span of 2 h in 15 min intervals.

### 2.7 Mass spectrometry for proteomics

Proteins were isolated from cell culture 48 h after siRNA treatment by lysing the detached and washed cells in 8 M Urea in TRIS‐Borat‐EDTA‐buffer (UT) via ten rounds of freeze thaw cycles in liquid nitrogen and on a heat block at 37°C. After centrifugation at 18000× *g* for 30 min the protein concentration in the supernatant was determined by BCA assay (7780S, New England Biolabs). Six micrograms of protein per replicate were used for tryptic digestion. Protein solutions were diluted in ammonium bicarbonate buffer (ABC), and sequencing grade modified trypsin (V5111, Promega) was used for digestion in a ratio of 1:25 at 37°C overnight. After stopping the reaction by adding acetic acid to a final concentration of 1% and spinning down at 18000× *g* for 30 min, the resulting peptides were purified by the PureSpeed C18 (Mettler‐Toledo GmbH) procedure. Samples were lyophilized, stored at −80°C, and resolved immediately before LC‐MS/MS analysis. Data were acquired on a Q Exactive instrument (Thermo Fisher Scientific) coupled with an Ultimate 3000 RSLC (Dionex). Raw data were processed for label free quantification (LFQ) with MaxQuant 1.6.2.10^16^ using a database of all reviewed human UniProt entries in revert decoy mode. Data were analyzed in separate batches for the cell line WM793b and 1205LU samples. Further parameters were set to a minimal peptide length of 7 amino acids, to variable modifications of Oxidation (M) and Deamidation (N) with maximum five modifications per peptide, to a maximum peptide mass of 9900 Da, to a matching time window of 0.7 min and an alignment time window of 20 min for matching between runs, to the digestion rule of Trypsin/P with not more than one missed cleavage, to MS1 tolerance of 0.5 Da, and to an FDR limit of 0.1. Quantitative protein abundance values were normalized on the sum of all intensities in the respective replicate run (Genedata Analys, GeneData Inc). Principle component analysis (PCA) was performed and replicates were checked for comparability by evaluating quantile‐normalized intensities of biological replicates by using Genedata Analyst (GeneData Inc).

### 2.8 Enrichment analysis

Effect size was calculated for protein intensities in the NRF2 knockdown dataset and in the KEAP1 knockdown dataset. Proteins with a log2 fold change above 1 or below −1 and a *p*‐value below 0.15 (716 in total) were selected for enrichment analysis carried out with the GSEA 3.0 software.^17^, ^18^ The analysis used the H: hallmark gene set (h:all.v6.2).^19^ Further settings included a weighted enrichment statistic, t‐test metric for ranking genes and a set size limitation between 15 and 500.

### 2.9 3D cell spheroid culture

Cells were seeded for 3D culture at a density of 3,000 cells per well in PrimeSurface^®^ 96 well low adhesion round bottom plates (MS‐9096UZ, PHC Corporation). Culture lasted between 48 and 96 h, with the culture medium being exchanged once 72 h after seeding. Images were taken every 24 h with a Leica DMI6000 B (Leica Camera). Maximum diameter was obtained from images by superimposing a circle over the image matching the outer edges of the spheroid. This evaluation was done in Adobe Photoshop (Adobe).

### 2.10 Cell treatment and viability assays

After overnight cultivation and following treatment with the respective substance of interest for 24 to 72 h, the medium was removed, and cells were lysed in 100 µl of CellTiter‐Glo^®^ Reagent (G7571, Promega). After 10 min of signal development, the luminescence signal intensity was acquired in a SpectraMax i3x Multi‐mode microplate reader (Molecular Devices). 3D cell spheroids were lysed in the round bottom culture plates by 100 µl of CellTiter‐Glo^®^ Reagent after 96 h of treatment. Relative cell viability was calculated by normalizing on the control condition of cells having undergone the same siRNA procedure but not receiving substance treatment and setting the non‐targeting siRNA condition as 100% basis. Vemurafenib (V‐2800, LC Laboratories) was resolved in dimethyl sulfoxide (DMSO, 34943, Sigma‐Aldrich) at a concentration of 2.5 mg/mL (5 mM). MYC inhibitor 10058‐F4 (F3680‐5MG, Sigma‐Aldrich) was resolved in DMSO at a concentration of 5 mg/mL (20 mM).

### 2.11 Correlation of gene expression in patient datasets

For correlating gene expression of identified genes of interest with marker genes for cell adhesion and invasion, the human skin cutaneous melanoma (SKCM) dataset, obtained from The Cancer Genome Atlas (TCGA) project, was utilized. Corresponding graphs were generated on the xenabrowser website (https://xenabrowser.net) or in GraphPad Prism 8.1 (Graphpad Holdings), respectively.

### 2.12 Patient samples and staining

The study was submitted to the Institutional Review Board of the IMC University of Applied Sciences (02.09.2015) and approval was waived for this study due to usage of retrospective and anonymized patient samples provided by BiomaxUS. Human tissue microarray slides (Me2082a, US Biomax) were warmed at 60°C for 30 min, deparaffinized using Roticlear (A538, Carl Roth) and rehydrated using a graded alcohol series. After dH_2_O washing, antigens were retrieved by bringing the slides to a boil in target retrieval solution (pH 6, S1699, Agilent Technologies). Then the slides were washed with PBS, incubated with 1% H_2_O_2_ for 15 min and permeabilized with 0.1% TritonX‐100/PBS for 5 min followed by washing and blocking with 1% BSA/PBS for 1 h and 1% BSA for 5 min. Next, the slides were incubated with the primary antibodies CD44 (1:600, 15675‐1‐AP, Proteintech Group) and NRF2 (1:200, ab62352, Abcam) at 4°C overnight. After washing, the slides were incubated with the biotinylated anti‐rabbit secondary antibody (1:500, BA‐1100, Vector Laboratories) at room temperature for 1 h. Then the slides were washed, incubated with Novocastra Streptavidin‐HRP (RE7104, Leica Biosystems) for 30 min, washed and incubated with aminoethyl carbazole (AEC) substrate (K3461, Agilent Technologies) for 9 min. After washing with dH_2_O, slides were counterstained with 1/3 diluted Mayer´s hemalum solution (1.09249, Sigma‐Aldrich) for 25 s and washed with tap water. Then the slides were mounted with Aquatex (1.08562, Merck Millipore) and after drying images were taken with the BX63 Intelligent Microscope (Olympus Corporation) under 20× magnification. Only tissues consisting of mainly tumor cells were used for further analysis. Percentages of AEC and hematoxylin (H) stained areas of the tissues were determined with the color deconvolution plugin of the software ImageJ 1.53c and resulting percentages were divided (AEC/H) and multiplied by 100 to acquire the staining intensity normalized to the number of nuclei.

### 2.13 Statistics

Statistics were done in GraphPad Prism 8.1 (Graphpad Holdings). Tests and *p*‐value ranges are indicated in the respective figure legends.

## 3 RESULTS

### 3.1 Targeting of NRF2 or its regulator KEAP1 by siRNA in melanoma cell lines

**FIGURE 1:**
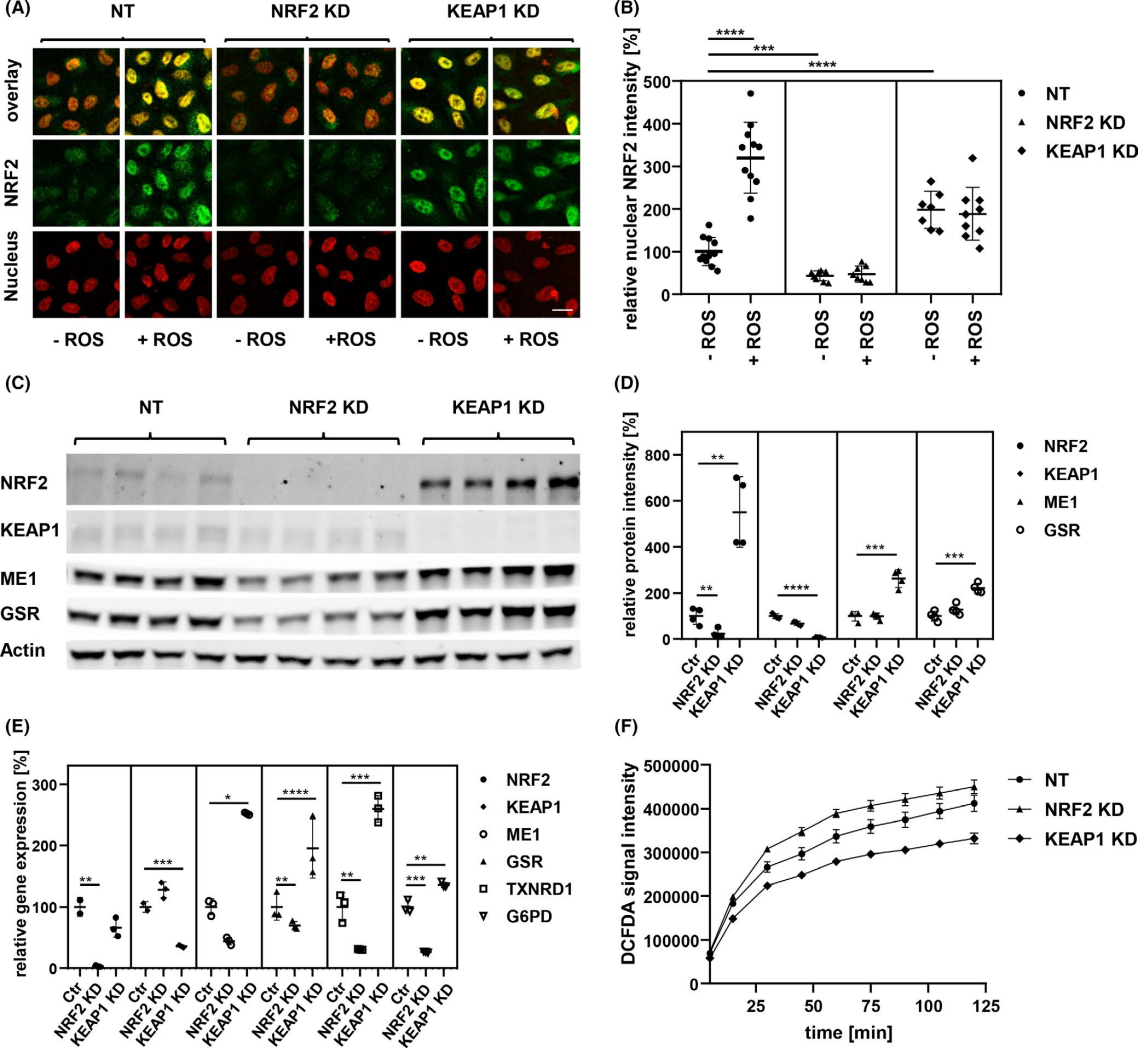
Melanoma cell lines were modified by siRNA to activate or inactivate the NRF2/KEAP1 regulation system. (A) Immunofluorescence images of WM793b cells with siRNA treatments, nucleus staining by DAPI, ROS stimulation with 5 µg/ml of Pyocyanin for 6 h, Scale bar: 10 µm. (B) Quantification of color intensity of NRF2 secondary antibody fluorescence in nuclear area, indicated by DAPI staining (unpaired *t*‐test; ***p‐value < 0.001; ****p‐value < 0.0001). (C) Western blot of WM793b protein extract 48 h after siRNA transfection, NRF2 and KEAP1 as siRNA targets, ME1 and GSR as targets of NRF2 transcription factor, β‐Actin as loading control. (D) Quantification of Western blot shown in C (unpaired *t*‐test; **p‐value < 0.01; ***p‐value < 0.001; ****p‐value < 0.0001). (E) qPCR of WM793b cDNA 24 h after siRNA transfection, NRF2 and KEAP1 as siRNA targets, ME1, GSR, TXNRD1 and G6PD as targets of NRF2 transcription factor (unpaired *t*‐test; *p‐value < 0.05; **p‐value < 0.01; ***p‐value < 0.001; ****p‐value < 0.0001). (F) ROS quantification by DCFDA fluorescence assay over 2 h with 10 mM Hydrogen peroxide (H_2_O_2_), in WM793b 48 h after siRNA transfection

We used four different melanoma cell lines, all harboring the frequent melanoma mutation BRAFV600E: WM793b, MCM1G, 1205LU, and MCM1DLN, the latter two show distant metastasis after transplantation to SCID mice.^14^, ^20^ All cell lines were used to knockdown either NRF2 or KEAP1 by siRNA in order to verify that control of NRF2 protein stability by KEAP1 is functional and hence establish a model for NRF2 induction or depletion. Exemplarily cellular localization of NRF2 is shown in WM793b cells (Figure 1A). Treatment with Pyocyanin, a strong ROS inducer, led to nuclear accumulation of NRF2, which was inhibited after NRF2 knockdown, while KEAP1 knockdown elevated basal NRF2 activity levels (Figure 1B). Western blot analysis of 1205LU cells showed successful knockdown (Figure 1C). Additionally, classical NRF2 target genes like ME1 and GSR were reduced upon NRF2 loss (now for clarity termed NRF2low cells), but increased after KEAP1 loss (termed NRF2high cells; Figure 1D). To further analyze target gene expression, we conducted quantitative real‐time PCR from reverse transcribed RNA. As expected ME1, GSR, TXNRD1, and G6PD showed significant decrease after NRF2 knockdown, but up‐regulation after KEAP1 knockdown (Figure 1E). Functional testing was carried out by measuring DCFDA fluorescence after incubating cells in culture medium for different periods of time (Figure 1F). Results for additional cell lines are shown (Figure S1). Overall, NRF2high cells showed lowest amounts of ROS, while NRF2low cells showed a strong increase at each time point. Our results showed that by modulating either NRF2 or KEAP1 in melanoma cells, the whole NRF2 system can be either blocked or enhanced. These findings enabled us to use this model in order to search for novel NRF2 targets in melanoma.

### 3.2 Proteome analysis of NRF2high versus NRF2low cells

**FIGURE 2:**
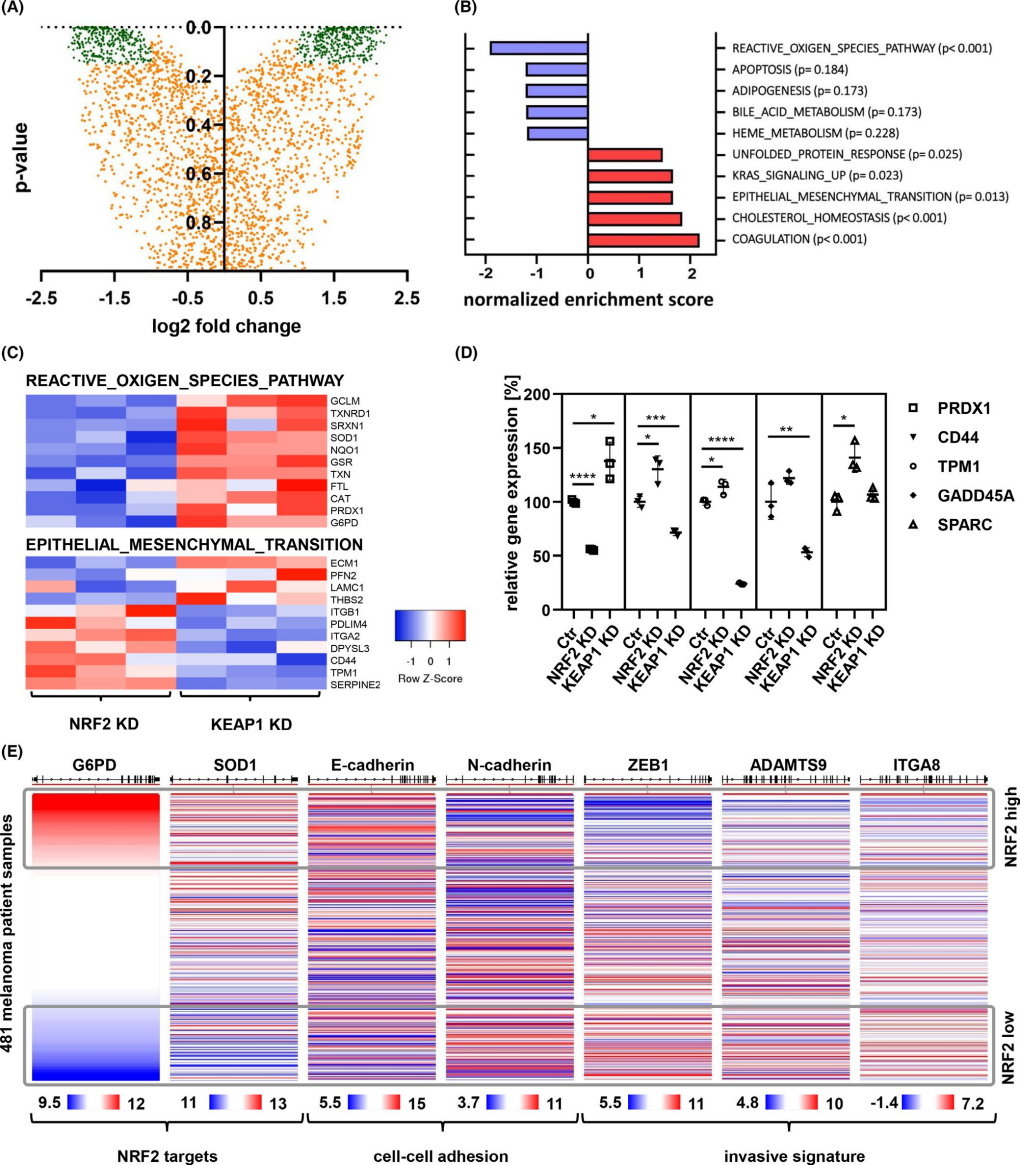
Shotgun proteomics of NRF2 modulated cells showed up‐regulation of malignancy markers upon NRF2 inactivation. (A) Protein expression changes in NRF2 reduced state (NRF2 knockdown vs KEAP1 knockdown, 1205LU), proteins used for hallmark identification (716) in green, with log2 fold change above 1 or below −1 and *p*‐value below 0.15. (B) Most significantly regulated hallmarks in NRF2 reduced state (NRF2 knockdown vs KEAP1 knockdown, 1205LU) from proteome dataset. (C) Heatmap of protein abundance for members of the ROS and EMT gene set. (D) qPCR of 1205LU cDNA 24 h after siRNA transfection. PRDX1 is a NRF2 target. CD44, TPM1, GADD45A and SPARC are members of the EMT hallmark set (unpaired *t*‐test; *p‐value < 0.05; **p‐value < 0.01; ***p‐value < 0.001; ****p‐value < 0.0001). (E) Display of NRF2 target genes together with cell adhesion and invasion markers in the TCGA human skin cutaneous melanoma data set. Expression is shown as log2(norm_count+1)

We used WM793b as well as 1205LU cells in triplicate to isolate whole cell proteins after siRNA‐mediated knockdown of NRF2 or KEAP1. Next samples were processed for lable‐free quantitative measurements by mass spectrometry. Quality of data was consistent across all samples and principal component analysis showed distinct grouping of samples after treatment (Figure S2A,B). Distribution of identified proteins in Nrf2 versus Keap1 siRNA treated cells is shown (Figure 2A). Furthermore, gene set enrichment analysis was performed with all available hallmark gene sets. Highest enrichment in NRF2high cells was the genset “Reactive_Oxygen_Species_Pathway”, while NRF2low cells showed enrichment in “Coagulation”, “Cholesterol_Homeostasis” and “Epithelial_Mesenchymal_Transition” (Figure 2B). Results for second cell line are shown (Figure S2C,D). NRF2 activity is known to be essential for regulating NADPH amounts. Disturbances in this balance were shown to impact the metabolic pathway of cholesterol homeostasis, which requires sufficient NADPH for synthesis.^21^ Most interesting to us was the identification of the EMT gene set, which shares 18 genes with the “Coagulation” gene set. Hence, we displayed key proteins involved in the ROS as well as in the EMT pathway (Figure 2C). Moreover, we validated the ROS‐induced gene PRDX1 and the EMT markers CD44, TPM1, GADD45A, and SPARC by real time PCR, which showed strong regulation as anticipated (Figure 2D). To further investigate the role of NRF2 we used the skin cancer cutaneous melanoma cohort established by TCGA. We asked whether low expression of the direct NRF2 targets G6PD and SOD1 would influence melanoma adhesion and invasion. Our analysis showed that EMT markers like N‐cadherin, ZEB1, ADAMTS9 and ITGA8, which were shown to regulate invasion in melanoma,^22^ where associated with low NRF2 target gene expression (Figure 2E).

### 3.3 Effects of MYC and BRAF inhibition in NRF2low cells

**FIGURE 3:**
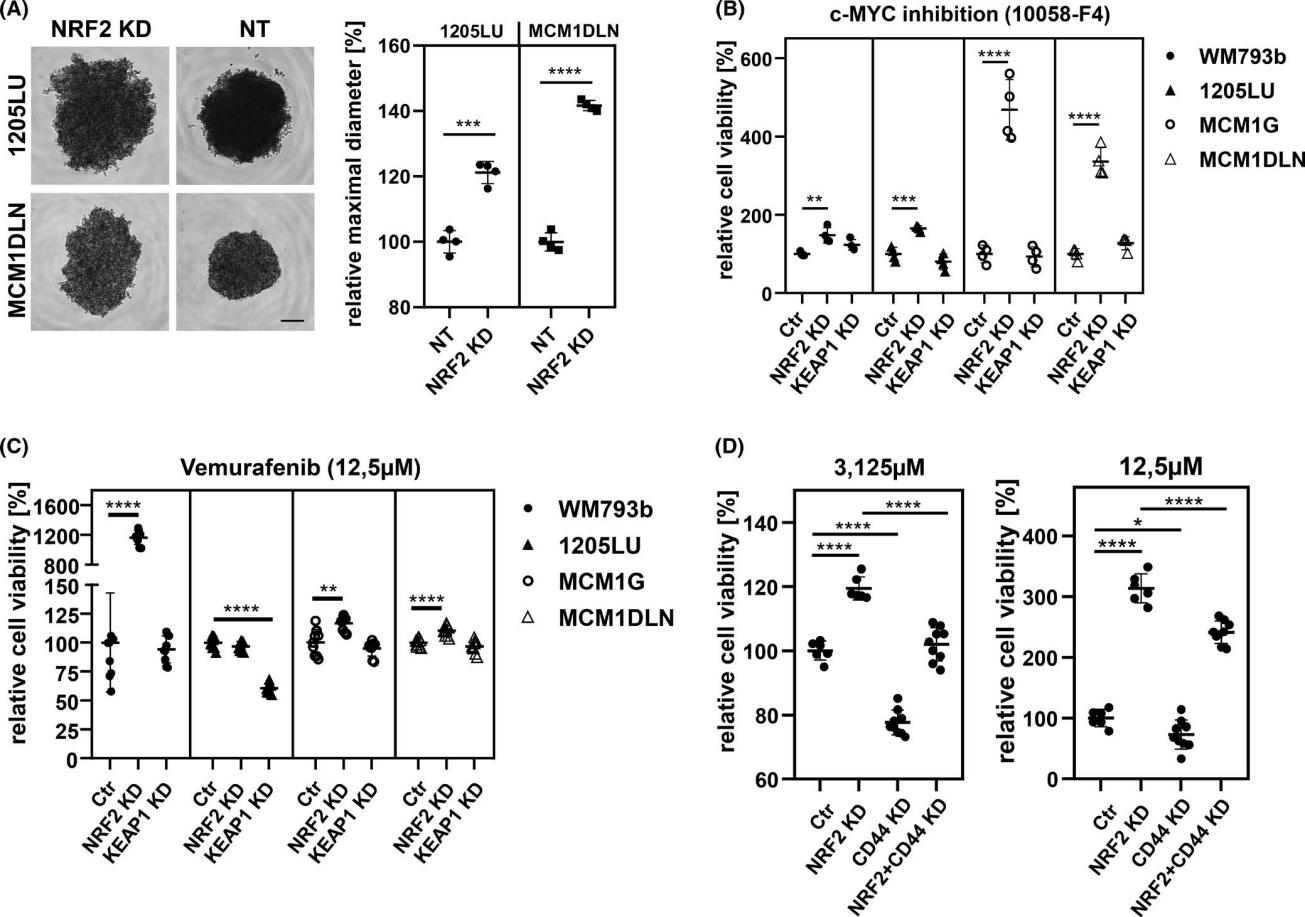
Cell lines with reduced NRF2 activity exhibited reduced cell‐cell adhesion tendencies and were less susceptible to inhibitor treatment. CD44 confers apoptosis resistance after vemurafenib treatment. (A) 3D cell spheroids forming in round bottom plate, 1205LU 48 h after seeding, MCM1DLN 72 h after seeding. Difference in diameter is an indicator of cell adhesion in 3D spheroids. Scale bar: 200 µm; (unpaired *t*‐test; ***p‐value < 0.001; ****p‐value < 0.0001). (B) Cell viability in spheroid culture relative to non‐targeting condition after 96 h treatment with c‐MYC inhibitor 10058‐F4 (25 mM) (unpaired *t*‐test; **p‐value < 0.01; ***p‐value < 0.001; ****p‐value < 0.0001). (C) Cell viability relative to non‐targeting condition after 72 h treatment with vemurafenib (12.5 µM) (unpaired *t*‐test; **p‐value < 0.01; ***p‐value < 0.001; ****p‐value < 0.0001). (D) Cell viability of WM793b cells relative to non‐targeting condition after 48 h treatment with Vemurafenib, after NRF2 and/or CD44 knockdown (unpaired t‐test; *p‐value < 0.05; **p‐value < 0.01; ***p‐value < 0.001; ****p‐value < 0.0001)

Tumor spheres are sophisticated cellular models to investigate cell‐cell interaction in three dimensional structures where inner cells are exposed to normoxia‐like environments. Hence, we tested the effects of NRF2 inhibition in this model. In control cells we observed formation of dense tumor spheres, which were non‐light permeable, whereas NRF2‐depleted cells were larger in diameter and less dense (Figure 3A). Adhesion of melanoma cells is mediated by various cadherins and integrins and for invasion and metastasis to occur cellular adhesion is down‐regulated.^23^ This process has been termed EMT and one of the drivers is the MYC transcription factor.^24^ Interestingly, the protein PRDX1, which we found down‐regulated in NRF2low cells, was shown to inhibit MYC target gene expression in NIH 3T3 cells.^25^ Hence, we next treated these tumor spheres with a pharmacologic MYC inhibitor (Figure 3B). Interestingly NRF2low cells showed significantly higher cellular viability after treatment. To further investigate the anti‐apoptotic effect of NRF2 inhibition we applied the clinically used BRAF inhibitor vemurafenib in BRAFV600E mutated cells. Three out of four NRF2low cell lines showed increased cell viability after treatment (Figure 3C). Still the unresponsive cell line showed strong decrease of viability when NRF2 was induced. Hence, we conclude that low levels of NRF2 increase viability while high levels of NRF2 have the potential to decrease viability. We used all cell lines and repeated the experiments also with a lower dose of vemurafenib. Additionally, removal of CD44 by siRNA showed that its expression is important for sustaining cell viability during vemurafenib treatment (Figure 3D). Control for knockdown of NRF2 and CD44 is shown (Figure S3A,B). Interestingly, vemurafenib survival‐advantage after NRF2 knockdown in 1205LU, MCM1G, and MCM1DLN was nearly completely depending on CD44 expression (Figure S3C–H).

### 3.4 CD44 and NRF2 expression in melanoma patient samples

**FIGURE 4:**
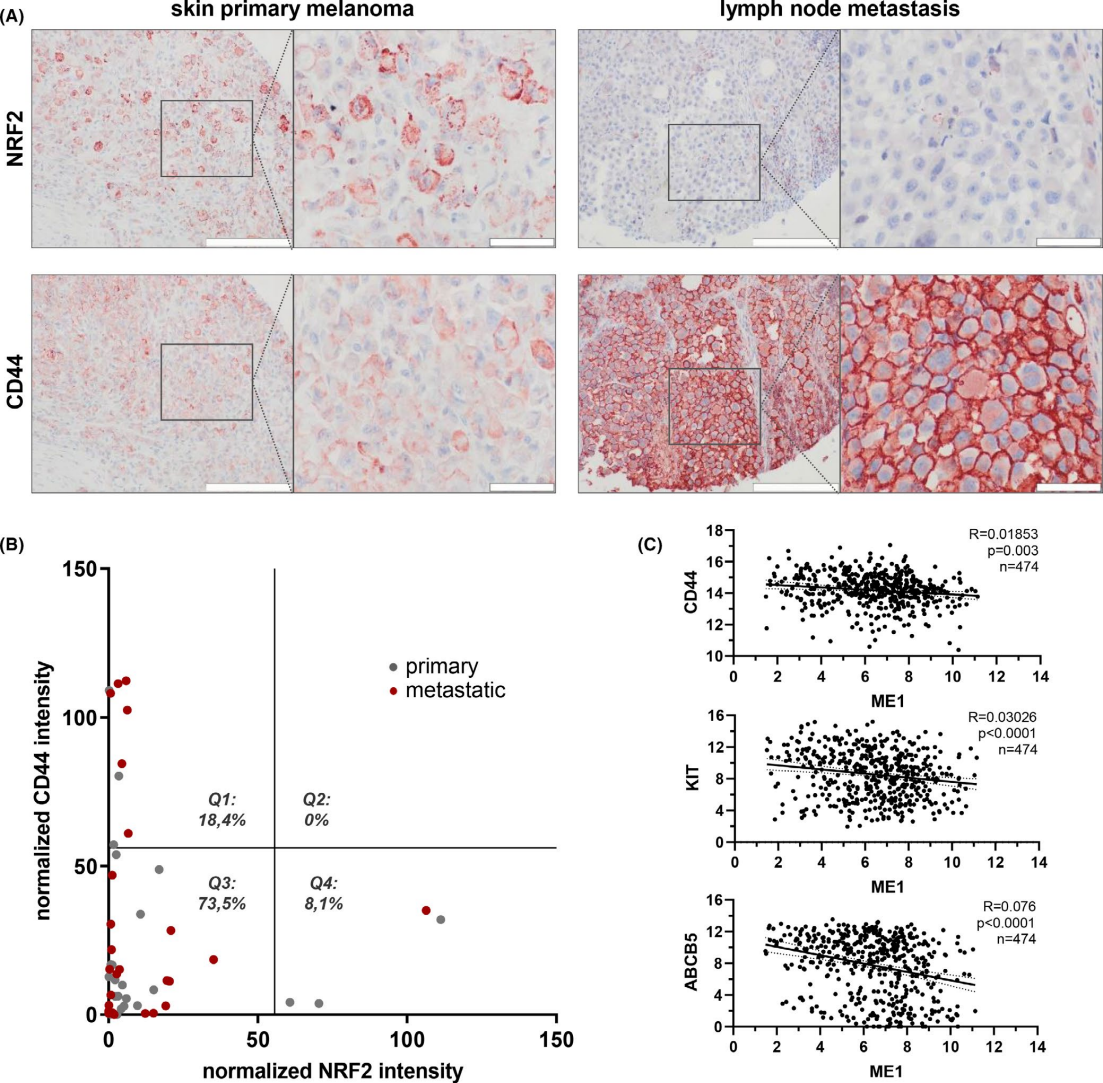
NRF2 and CD44 showed a vice versa expression pattern in melanoma patients. (A) Histologic stain of representative, consecutive melanoma tissue sections for NRF2 and CD44. Positive staining shown in red (AEC), nuclei in blue (hematoxylin) color. Scale bars: 20× = 200 µm, 60× = 50 µm; (B) AEC and hematoxylin signals of the stained tissue array slides were analyzed with the software ImageJ and are shown as NRF2 and CD44 staining intensity normalized to the number of nuclei. Grey dots represent tissues of primary melanomas, red dots represent tissues of metastatic melanomas. The graph was quartered at the halves of the measured NRF2 and CD44 intensities to gain quarters 1–4 (Q1–4). Only tissues containing mostly tumor cells were used for the analysis. *N* = 49 (C) Correlation of NRF2 target gene ME1 with marker genes, expression levels derived from the TCGA human skin cutaneous melanoma dataset (linear correlation, confidence interval 95%, non‐zero test for slope)

To elaborate on our findings, we investigated a collection of melanoma samples comprising primary tumors as well as metastatic samples. Sample cohort consisted of 26 males with ages between 44 and 80 years and 23 females with ages between 16 and 79 years. We used validated anti‐CD44 and NRF2 antibodies in order to quantify staining intensities on consecutive tissue slides (Figure 4A). Signal strength of all samples was calculated using ImageJ software with identical cutoff values for each antibody. Signal was normalized to nuclear staining of tumor tissue. We displayed the distribution of NRF2 versus CD44 staining intensity (Figure 4B). While 18.4% of all samples were solely CD44 positive in quadrant 1 (Q1), none were CD44 and NRF2 double positive (Q2) and 8.1% were solely NRF2 positive (Q4). 73.5% showed low expression of both markers (Q3). Whole, consecutive stains of six individual patient samples are also shown (Figure S4). Patient sample analysis showed that strong co‐expression of both markers could not be detected. Instead, individual samples were either CD44 or NRF2 positive. This supports the idea that tumor cells in vivo reside in distinct redox states, and it is interesting to note that from nine high CD44 and low NRF2 stained samples a total of six samples were derived from melanoma metastasis. Since CD44 is associated with invasion as well as with stemness, we decided to further elaborate on the effect of low NRF2 activity on KIT, a melanocyte stem cell marker^26^ and ABCB5, a melanoma stem cell marker.^27^ We analyzed data from the skin cutaneous melanoma cohort and could identify significant negative correlation between the NRF2 target malic enzyme 1 (ME1) and KIT as well as ABCB5 (Figure 4C).

**FIGURE 5:**
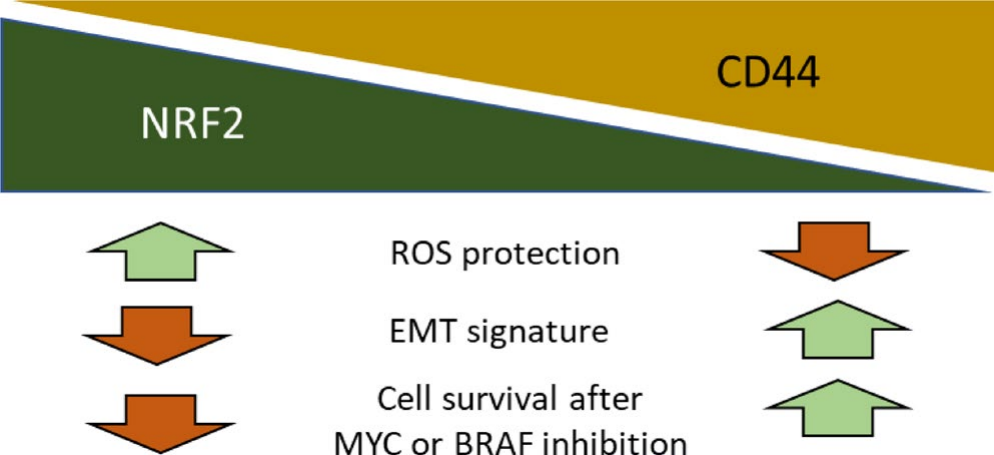
Schematic for the proposed role of NRF2 in melanoma. Loss of NRF2 led to an epithelial mesenchymal transition (EMT) phenotype and an up‐regulation of the stem cell marker CD44. Additionally, cells devoid of NRF2 showed increased cell viability after treatment with a pharmacologic MYC and a mutant BRAF inhibitor, but lost their protection against reactive oxygen species (ROS)

In summary, we identified a novel phenomenon after down‐regulation of NRF2. Cells entered an EMT‐like state, and showed increased survival after pharmacologic inhibition of MYC or mutant BRAF (Figure 5). CD44 receptor expression may represent a novel mechanism for melanoma cells to escape cellular stress conditions.

## 4 DISCUSSION

NRF2 serves multiple roles during tumor development. Here we have analyzed the effects of NRF2 induction versus NRF2 depletion in human melanoma cell lines. We could demonstrate that NRF2 activity up‐regulates proteins important for redox homeostasis and protects cells from ROS accumulation. Surprisingly, our proteome analysis showed that reduction of NRF2 levels promoted an EMT phenotype, which comprised up‐regulation of EMT associated genes like CD44, TPM1, GADD45A, and SPARC.

The process of EMT is rooted in developmental pathways, which, for example, determine the formation of neural crest cells after delamination from the neural tube during embryo formation.^28^ In adult tissue, high oxidative stress can also trigger EMT and interestingly NRF2 was shown to inhibit this form of EMT in renal cells as well as in lung tissue.^29^, ^30^

Recently it became clear that EMT in melanoma cells also plays a central role for establishing an invasive and metastatic phenotype.^31^ Furthermore, the presence of EMT has been linked to a tumor stem cell phenotype, which is hallmarked by high p75NGFR expression.^32^ Importantly the stem cell characteristics are accompanied by an insensitivity towards pharmacological BRAF inhibition. These results link the establishment of an EMT phenotype with increased resistance towards tumor therapy.

Our identification of CD44 up‐regulation as a consequence of NRF2 loss complements the aforementioned findings. When we induced cell death, either by MYC or pharmacologic BRAF inhibition, we monitored increased cellular survival after NRF2 loss. Furthermore, upon vemurafenib induced apoptosis we could show that CD44 expression was essential for mediating this effect. Therefore, we speculate that siRNA mediated NRF2 down‐regulation leads to the acquisition of a more apoptosis resistant melanoma cell either by adoption of specific stem cell functions or by an increase of anti‐apoptotic signaling. Interestingly, CD44 was one of the first identified cancer stem cell markers, indicative for tumor self‐regeneration after transplantation into immune‐deficient mice.^33^ CD44 binds extracellular hyaluronan and activates effectors like RHO, RAC1, and RAS, leading to mitogen activated protein kinase and PI3 kinase activation.^13^ Additionally, CD44 expression can lead to receptor tyrosine kinase signaling, including transforming growth factor beta receptor type 1, and hence it has been associated with cancer cell chemoresistance.^34^ Furthermore, beyond its constitutively expressed region, CD44 has a number of variable exons. Our study here is focused on the CD44 standard form, analysis of the variant form has so far turned out very challenging.^35^, ^36^

Melanoma cells harbor the possibility to alter their cellular phenotype from growth and proliferation to cell cycle arrest and migration by performing a cellular switch. This so‐called phenotype switching is regulated by transcription factors like MITF as a prominent member, controlling tumor growth, cell division and cell differentiation. Recently, it was shown that NRF2 inhibits action of MITF.^37^ Hence, it is plausible that also in our experiments, induction of NRF2 reduced MITF amounts, while depletion of NRF2 induced MITF activity. MITF itself is a master regulator of melanocyte differentiation, has anti‐apoptotic properties in melanoma and increases cellular survival.^33^ Thus, down‐regulation of NRF2 not only induces CD44, but maybe also MITF, and both markers confer anti‐cytotoxic abilities to melanoma cells.

Concluding, NRF2 has pro‐tumorigenic roles in cancer and it is tempting to therapeutically inhibit NRF2 function in melanoma. However, we have shown that inhibition of NRF2 up‐regulates CD44 and that this molecular mechanism could be detrimental to the therapeutic outcome of NRF2 inhibition. To the best of our knowledge, there are currently no clinical trials ongoing, which target NRF2 in order to fight melanoma growth. Still, we want to raise awareness when developing and planning NRF2 intervention therapies to consider the presented consequences of NRF2 loss in melanoma. Future approaches to inhibit NRF2 in melanoma should be combined with CD44 targeting and thus improve efficacy and clinical outcome.

## CONFLICT OF INTEREST

The authors declare no conflict of interests.

## AUTHOR CONTRIBUTIONS

Conceptualization: Mikula M.; Methodology and data acquisition: Weitzenböck H.P., Gschwendtner A., Wiesner C., Depke M.; Review and interpretation of data: Wiesner C., Schmidt F., Trautinger F., Hengstschläger M., Hundsberger H., Mikula M.; Writing and editing: Weitzenböck H.P. Gschwendtner A., Mikula M.; Project administration: Hundsberger H., Mikula M.; Supervision: Mikula M. All authors have read and agreed to the published version of the manuscript.

## ETHICAL APPROVAL STATEMENT

Anonymized human melanoma tissue was obtained from the company US Biomax. The study was approved 05/11/2015 by the Institutional Research Ethics Board of the Danube University Krems.

## Supporting information

Fig S1‐S4

## Data Availability

Data that supports the findings of this study are available from the corresponding author upon request.
