# Supplementary material for: Proteome analysis of NRF2 inhibition in melanoma reveals CD44 up‐regulation and increased apoptosis resistance upon vemurafenib treatment
Source: Cancer Med. 2021 Dec 23;11(4):956–67. doi: 10.1002/cam4.4506 (PMC8855890; doi:10.1002/cam4.4506)
Supplement: Supplementary file 1 — Fig S1‐S4 [file CAM4-11-956-s001.pdf]

Supplementary Figure S1

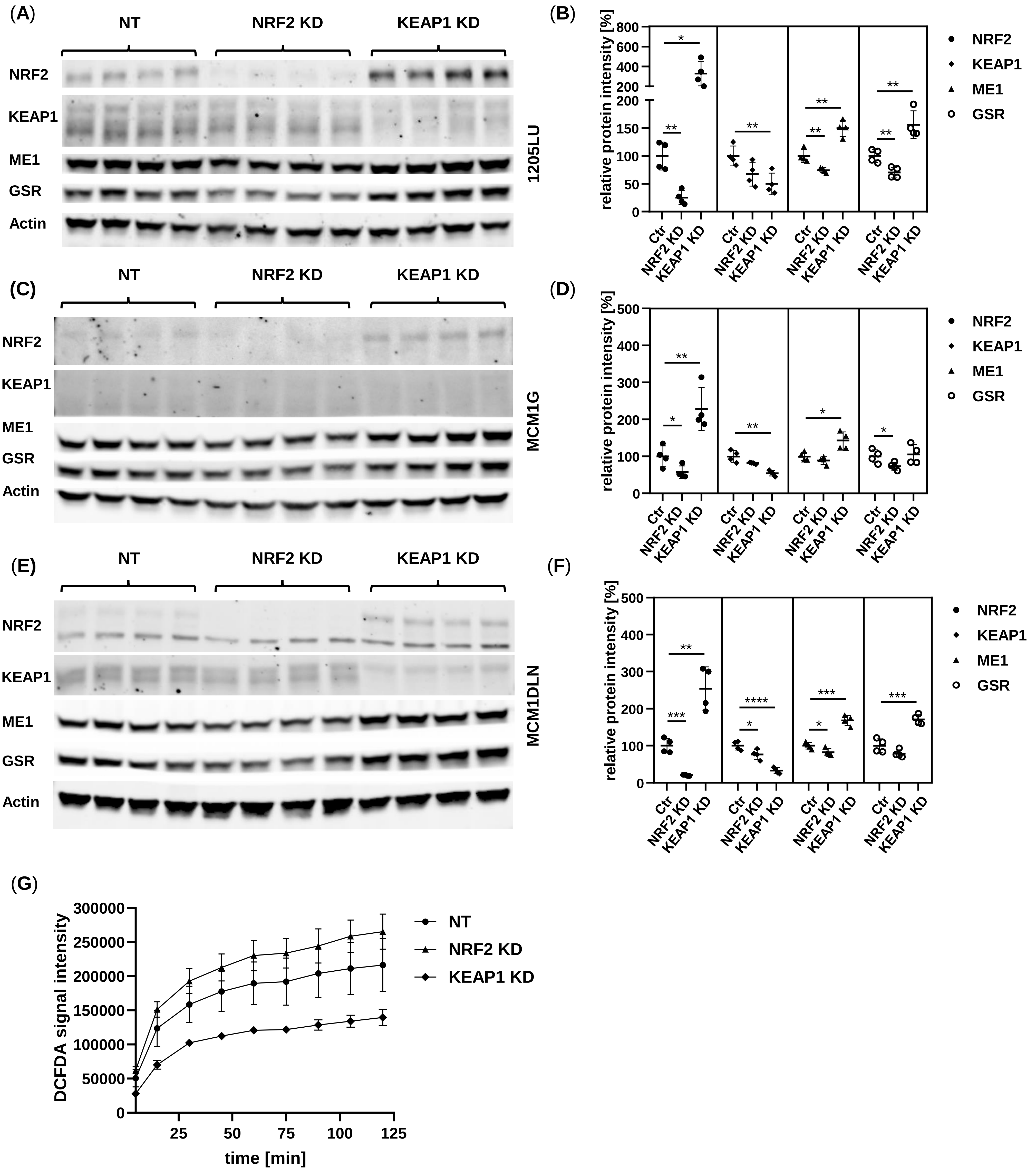

Figure S1. Western blots of protein extract from siRNA modulated cells. **(A)** Western blot of 1205LU protein extract 48 hours after siRNA transfection, NRF2 and KEAP1 as siRNA targets, ME1 and GSR as targets of NRF2 transcription factor, β-Actin as loading control. **(B)** Quantification of Western blot shown in A (unpaired t-test; * p-value < 0.05; ** p-value < 0.01; *** p-value < 0.001; **** p-value < 0.0001). **(C)** Western blot of MCM1G protein extract 48 hours after siRNA transfection. **(D)** Quantification of Western blot shown in C. **(E)** Western blot of MCM1DLN protein extract 48 hours after siRNA transfection. **(F)** Quantification of Western blot shown in E. **(G)** ROS quantification over 2 hours with 10mM Hydrogen peroxide (H_2O_2), in 1205LU 48 hours after siRNA transfection.

Supplementary Figure S2

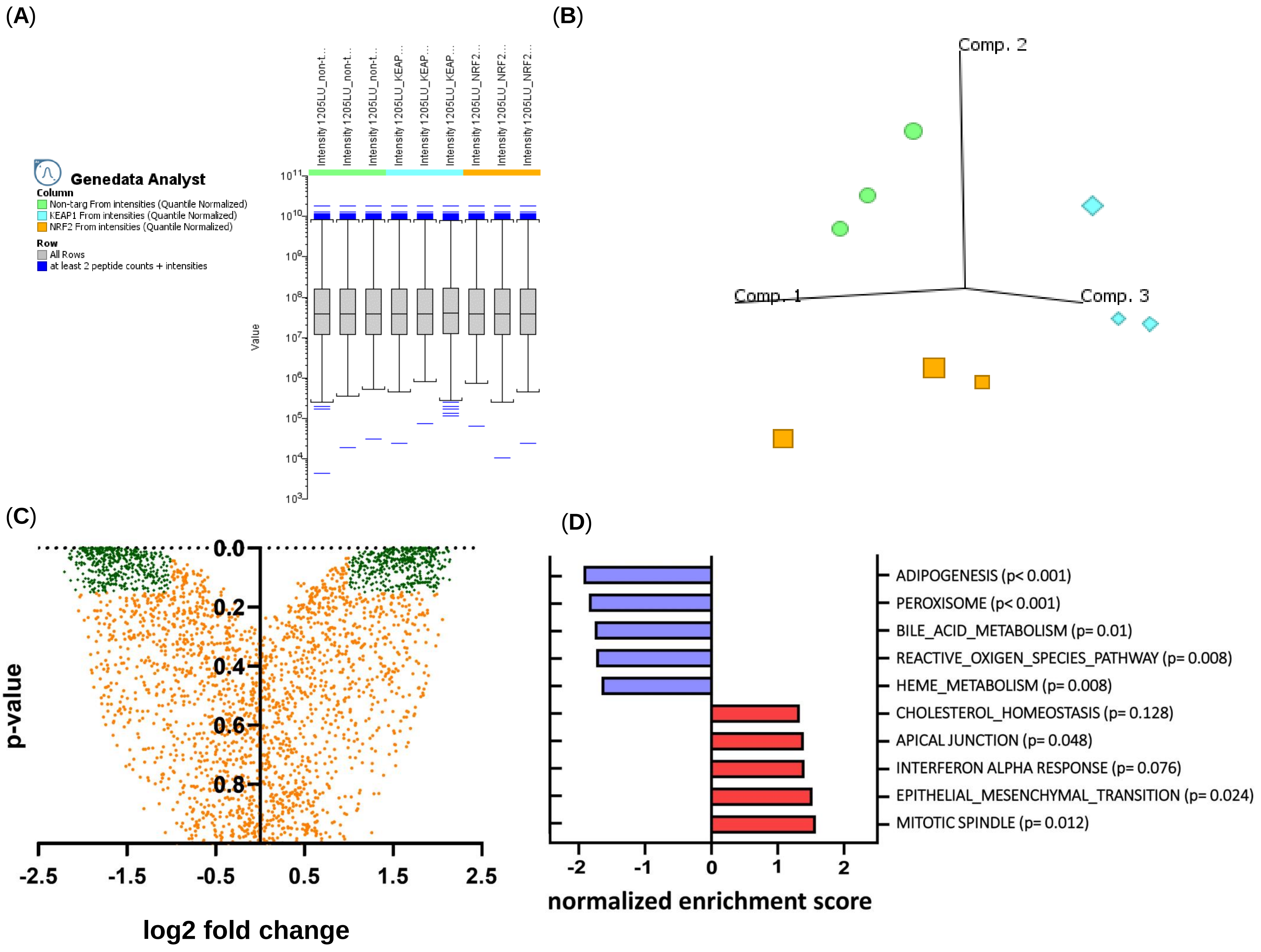

Figure S4. Quality control plots for proteomics analysis of 1205LU cells and enrichment analysis of proteome data generated from WM793b cells. **(A)** Quantile normalized intensity of biological replicates from siRNA treated 1205LU cells, with at least 2 peptide counts. **(B)** Principal component analysis of biological replicates from siRNA treated 1205LU cells. **(C)** Protein expression changes in NRF2 modulated state (NRF2 knockdown vs KEAP1 knockdown, WM793b), proteins used for hallmark identification (891) in green in green, with log2 fold change above 1 or below -1 and p-value below 0,15. **(D)** most significantly regulated hallmark genesets in NRF2 modulated state (NRF2 knockdown vs KEAP1 knockdown, WM793b) from proteome dataset.

Supplementary Figure S3

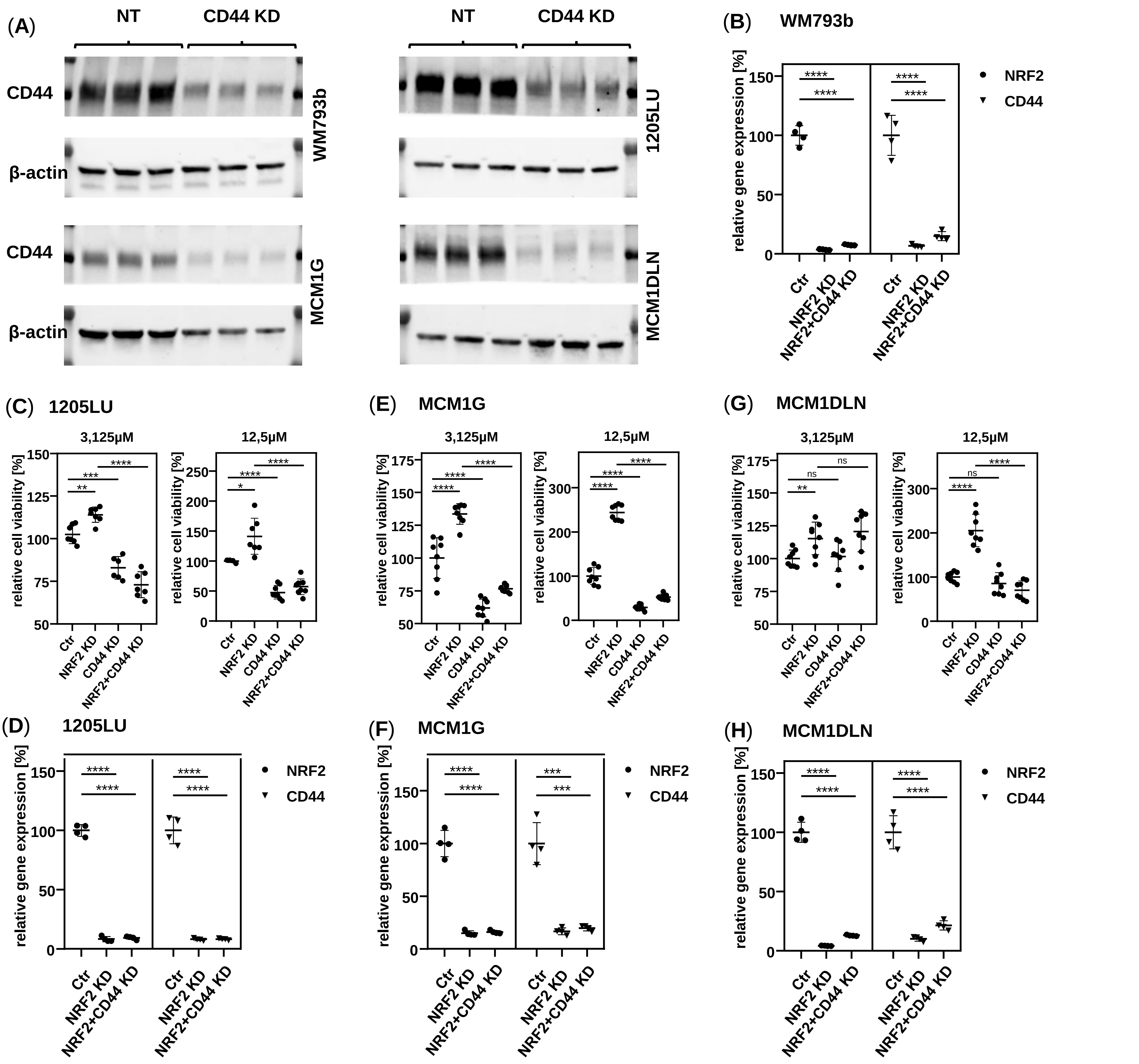

Figure S3. Confirmation of CD44 and NRF2 combined knockdown and effect of NRF2 on cell survivability upon Vemurafenib treatment. **(A)** Western blot of protein extract 48 hours after siRNA transfection, primary antibodies against CD44 as siRNA target and β-actin as loading control. **(B)** qPCR of WM793b cDNA 24 hours after siRNA transfection, NRF2 and CD44 as (combined) siRNA targets (unpaired t-test; *** p-value < 0.001; **** p-value < 0.0001). **(C)** Cell viability of 1205LU cells relative to non-targeting condition after 48 hours treatment with Vemurafenib, after NRF2 and/or CD44 knockdown (unpaired t-test; ns p-value > 0.05; * p-value < 0.05; ** p-value < 0.01; *** p-value < 0.001; **** p-value < 0.0001). **(D)** qPCR of 1205LU cDNA 24 hours after siRNA transfection, NRF2 and CD44 as (combined) siRNA targets. **(E)** Cell viability of MCM1G cells relative to non-targeting condition after 48 hours treatment with Vemurafenib, after NRF2 and/or CD44 knockdown. **(F)** qPCR of MCM1G cDNA 24 hours after siRNA transfection, NRF2 and CD44 as (combined) siRNA targets. **(G)** Cell viability of MCM1DLN cells relative to non-targeting condition after 48 hours treatment with Vemurafenib, after NRF2 and/or CD44 knockdown. **(H)** qPCR of MCM1DLN cDNA 24 hours after siRNA transfection, NRF2 and CD44 as (combined) siRNA targets.

Supplementary Figure S4

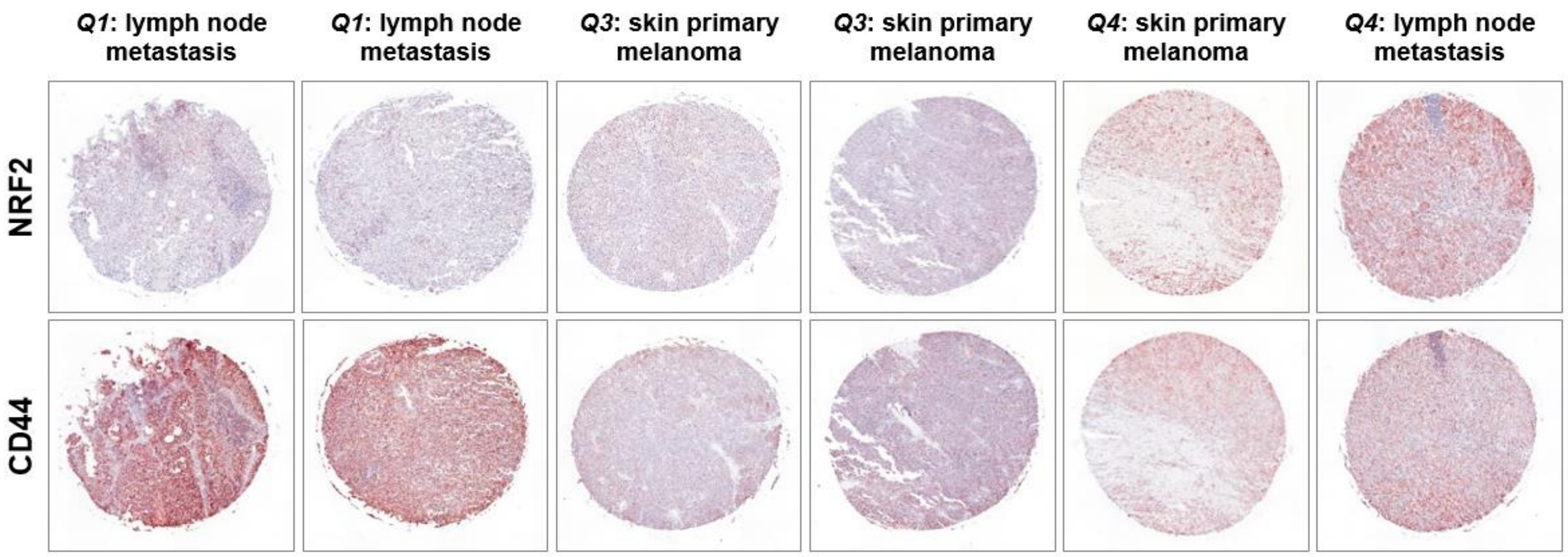

Figure S4. Consecutive tissue array slides of melanoma patient samples were immuno-stained for NRF2 and CD44 by AEC (red color). Whole tissue sample pictures of representative NRF2 (top) and CD44 (bottom) stained tissues of Q1 (lymph node metastases), Q3 (skin primary melanomas) and Q4 (skin primary melanoma and lymph node metastasis) are shown.
